# Host determinants of HTLV-1 integration site preference

**DOI:** 10.1186/1742-4690-12-S1-O27

**Published:** 2015-08-28

**Authors:** Amy J McCallin, Goedele N Maertens, Charles RM Bangham

**Affiliations:** 1 Division of Infectious Diseases, St Marys Hospital, Imperial College London, W2 1PG, UK

## 

Integration of the provirus into the host genome is an essential step in the establishment of a chronic retroviral infection. Retroviruses do not integrate at random. Each genus of retrovirus preferentially integrates in genomic sites associated with certain genomic features. This preference is thought to result from an interaction of the pre-integration complex with a specific host co-factor. Such an interaction has been well documented between HIV-1 integrase and lens epithelium-derived growth factor (LEDGF) and between MLV and members of the Bromoextraterminal Domain Protein (BET) family. HTLV-1 also has a reproducible preference to integrate near various genomic features; however, the putative host co-factor of HTLV-1 integrase remains unknown. We present evidence that demonstrates that the HTLV-1 integrase co-factor may be the protein phosphatase 2A (PP2A) complex. Two independent protein interaction screens (yeast-2-hybrid and co-immunoprecipitation) both revealed an interaction between HTLV-1 integrase and the PP2A holoenzyme. We used co-culture with HTLV-1 producer cell line MT2 to infect rat cell lines containing an inducible knockdown of PP2A. We found that PP2A-knockdown altered the integration profile of these de novo integration sites. In contrast to infection in PP2A-competent cells, integration sites in PP2A-knockdown cells lacked the characteristic proximity to genomic features associated with transcriptional activity (genes, transcription start sites, CpG islands) and to epigenetic marks associated with active chromatin. We are in the process of investigating how PP2A inhibitors and shRNAs targeting specific regulatory subunits of the PP2A holoenzyme influence integration site selection.

